# False Teeth

**Published:** 1880-08

**Authors:** 


### EDITORIAL, ETC.

*False Teeth.*—At the meeting of the National Dental Association held in New York City during the present month, Dr. Patrick, president, made a speech on the subject of the business of dentistry, its growth and importance, which cannot fail to have a depressing effect upon those who speculate on the infirmities of poor human nature. There are, he said, 12,000 dentists in the United States—that is to say, one to every 3,000 men, women and children in the country—and these dentists must make a living by drawing, inspecting, repairing and supplementing our defective teeth. They annually pack into our unsound teeth not less than half a ton of pure gold, worth $500,000, besides four times as much cheaper material—silver and platinum—worth $100,000 more. Conceive the American people going around with two tons and a-half of metal in their mouths! It was also a fact, said Dr. Patrick, that three million false teeth were made and planted in our mouths every year. Think of it! The present population of the country between the ages when the second teeth have grown and that when teeth cease to be an object is less than 54,000,000, requiring, in a full dentition, which scarcely any have, 768,000,000 teeth. Allowing an average of 20 teeth to the head we have 480,000,000 teeth. The average duration of a false tooth may be put at ten years, so that there must be now in this country not less than 30,000,000 false teeth—one in every sixteen. This is independent of the patched and repaired teeth, and the numerous "snags" which are not worth looking after. The worst of it is that Dr. Patrick thinks our teeth tend to grow worse instead of better. The rule of the "survival of the fittest" does not work in this case. The perfect set of (false) teeth in this generation does not become the perfect set of natural teeth in the next. On the contrary, Dr. Patrick has no hesitation in saying "that the next generation will witness a greater abnormality in dental development than we have yet seen. It is for this simple reason that there is a greater tendency to nervous and cerebral diseases resulting from fast living and injudicious food. The Doctor advises the dentists to rise to a higher plane to meet the coming disease.

---

### MONTHLY SUMMARY.

